# Whispering galleries and the control of artificial atoms

**DOI:** 10.1038/srep25084

**Published:** 2016-04-28

**Authors:** Derek Michael Forrester, Feodor V. Kusmartsev

**Affiliations:** 1 Loughborough University, Departments of Chemical Engineering and Physics, Loughborough, LE11 3TU, United Kingdom; 2 Loughborough University, Department of Physics, Loughborough, LE11 3TU, United Kingdom

## Abstract

Quantum computation using artificial-atoms, such as novel superconducting circuits, can be sensitively controlled by external electromagnetic fields. These fields and the self-fields attributable to the coupled artificial-atoms influence the amount of quantum correlation in the system. However, control elements that can operate without complete destruction of the entanglement of the quantum-bits are difficult to engineer. Here we investigate the possibility of using closely-spaced-linear arrays of metallic-elliptical discs as whispering gallery waveguides to control artificial-atoms. The discs confine and guide radiation through the array with small notches etched into their sides that act as scatterers. We focus on *π*-ring artificial-atoms, which can generate their own spontaneous fluxes. We find that the micro-discs of the waveguides can be excited by terahertz frequency fields to exhibit whispering-modes and that a quantum-phase-gate composed of *π*-rings can be operated under their influence. Furthermore, we gauge the level of entanglement through the concurrence measure and show that under certain magnetic conditions a series of entanglement sudden-deaths and revivals occur between the two qubits. This is important for understanding the stability and life-time of qubit operations using, for example, a phase gate in a hybrid of quantum technologies composed of control elements and artificial-atoms.

Nanoscale defects and notches in microlaser devices lead to confinement effects and redirected emission. In designing microlasers problems arise primarily due to low efficiency and uncontrolled scattering events^1^. With this in mind a new generation of microlasers that break rotational symmetry and deliberately include wavelength sized notches has solved the problem of low power collection and channelling the emission^2^. Assemblies of chains of microlasers have many prospective applications in computing, solar harvesting^3^ and also biological sensing^4^.

Electrons in metals readily respond to electromagnetic stimulation, with especially remarkable results for sub-wavelength dimensioned structures^5^. An electromagnetic response can also be induced in a multitude of materials, including those that are non-magnetic in isolation^6^, when geometries are patterned smaller than the incident fields wavelength (e.g.^7^,^8^,^9^,^10^). Using external fields to exert control over quantum devices has been actively researched for some years^11^. In this research we show that defects engineered into microlasers can control the quantum entanglement between two superconducting *π*-rings or analogously nanomagnets or any such system that is composed of four clearly definable quantum states. In the following we will study the use of quantum gates to control the level of entanglement for two qubits. The measure of entanglement employed for two qubits is called concurrence. Quantum entanglement is of central importance for functional quantum devices. Using the principles behind concurrence Yu and Eberly^12^ discovered that entanglement may be born out of a bipartite system only to endure a series of deaths interspersed by revivals. This phenomenon was named entanglement sudden death (*ESD*)^13^. We find that this occurs for certain parameters in a system of two coupled flux qubits that are controlled by a plasmonic waveguide. The first demonstration of controllable coupling of flux qubits occurred in 2007^14^ through an additional coupler loop, with the development building on earlier discoveries of entanglement between two of this type of qubit^15^. Here the control is performed through the plasmonic waveguide that is composed of two lines of elliptical disks as shown schematically in Fig. 1 (other architectures are also shown schematically in the supporting information). Artificial atoms, such as superconducting qubits are solid-state analogues of optical beam splitters and interferometers and have been discussed in the context of tight–binding networks too^16^.

It is an interesting but natural progression for quantum technologies to become hybrids of different systems^17^. We present the example of a system of coupled qubits that could be linked by plasmonic structures. If we have an open system then it implies a coupling to the environment. The surface of the system supports plasmon modes and can provide a very sensitive mediation of the interaction between the qubits. Flux qubits are highly sensitive to the changes in magnetic energy in the system. Thus, in the foreseeable future plasmonic metamaterials will mediate the interaction between superconducting rings, disks and other magnetic systems. In superconducting systems these will operate at microwave and terahertz frequencies. Our system can consist of any magnetically responsive quantum device to be controlled by the waveguides. For example, one very favourable design of qubit is a ring of superconducting islands interspersed by Josephson junctions. Of these superconducting qubits perhaps the most robust and promising is the flux qubit. Even more appealing is the *π*-ring qubit which can generate its own orbital moment^18^,^19^,^20^,^21^,^22^,^23^,^24^,^25^. With this feature the need for external control circuitry becomes minimal. The spontaneous-currents of the *π*-rings may flow clockwise or anti-clockwise, giving us the quantum states 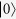 and 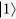, respectively. The two inductively coupled flux qubits, with two-state pseudo-spins that are related to the phase differences over the Josephson junctions, are highly sensitive detectors of magnetic field changes.

## Results

### Micro-disk control elements

The two superconducting *π*-rings can be thought of as two artificial atoms (controllable over 1–8 *THz* which is also the frequency range of important biomolecules^26^), interacting with mutual inductance if close enough to one another and manipulated by the magnetic field generated from the plasmonic array. Indeed, superconducting quantum systems have previously been designed as macroscopic “articial atoms” coupled with transmission line architectures to carry out photonic quantum information processing^27^,^28^. In the proposed plasmonic system, by directing an electromagnetic wave at an edge elliptical cone a static magnetic field oriented in the *z* direction manipulates the state of the system in air. The simplest interaction between surface plasmon supporting structures is perhaps two dots of circular or elliptical geometries. In these structures the applied electromagnetic field can be greatly enhanced and whispering galleries can be obtained. It then becomes an issue of engineering the structures out of materials with low losses and appropriate indices of refraction. We demonstrate two lines of coupled ellipses. The plasmons that propagate from one dot to the next are characterised by a subwavelength confinement of the electromagnetic field. The propagation can occur over a long range without detrimental losses. Usually metamaterials are made from subwavelength patterns of metallic and dielectric fillers or substrates. For conventional metals there are high losses that give limitations for their use in metamaterials. For this reason there is a plethora of designs based upon using noble metals, i.e. they have comparatively smaller losses to most other metals. But they are far from ideal materials and tunability of the large negative permittivity magnitudes is not really possible. The order of the permittivity constant would ideally approach the magnitude of the dielectric constant in a metamaterial design. So the objective in plasmonics is engineering new materials with optimal electromagnetic responses. For this reason low loss superconducting devices^29^ are very desirable and can themselves operate as plasmonic metamaterials^30^. However, we focus on the terahertz region of the electromagnetic spectrum as a natural frequency range for controlled plasmonics of Josephson junction metamaterials and qubits^30^,^31^,^32^. In designing the control network for channelling radiation into quantum bits or devices, with components that become close in size to the wavelength of propagation, electromagnetic fields are blocked by diffraction limitations and hence there is a critical dimension associated with the minimal size of optical structures, even in the case of photonic crystals^33^. This is where plasmonics fills in this technology gap. In surface plasmonics electromagnetic waves are trapped as they resonantly interact with plasmas of electrons and sub-wavelength devices become possible. This resonant structure can be used as a plasmonic waveguide. We use small dots of different geometries rather than larger waveguides as they can pass information between one another (as in Fig. 2) with a reduced metallic volume and hence a notable reduction in ohmic losses, in contrast to when using conventional plasmonic metals. The idea is to use plasmonic arrays to mediate the electromagnetic interaction of the qubits, to create entanglement and manipulate the state of the system whilst providing a modicum of protection from environmental noise. Using plasmonics one can also manipulate electromagnetic radiation over a broad range of frequencies. In Fig. 2 there is a small defect in the left hand elliptical structure which traps an incoming electric field. This is of vital importance and it is with the use of defect engineering that surface plasmonics can reach its true potential. For example, in experiments using plasmonic arrays for surface enhanced Raman spectroscopy (*SERS*), it seems little understood why enhancement effects are sometimes seen and at others not. The most straightforward reason is due to surface effects and localised confinements that are highly dependent upon frequency and losses. When an electromagnetic wave strikes a surface, small defects can completely change the propagation through the system. Gonzalez-Tudela *et al*.^34^ used a plasmonic waveguide to control the entanglement of qubits. In their case they entangled two qubits through a 1D plasmonic wave-channel. The method entails tracing out the degrees of freedom of the plasmons in the system through a master equation for the density matrix^17^. Here we will control a phase gate with a system comprised of electronically isolated control elements and qubits. In the supporting information a density matrix analysis is also given, demonstrating the sensitivity of entanglement to temperature and system energy balances.

### Two qubit entanglement

**Figure 1:**
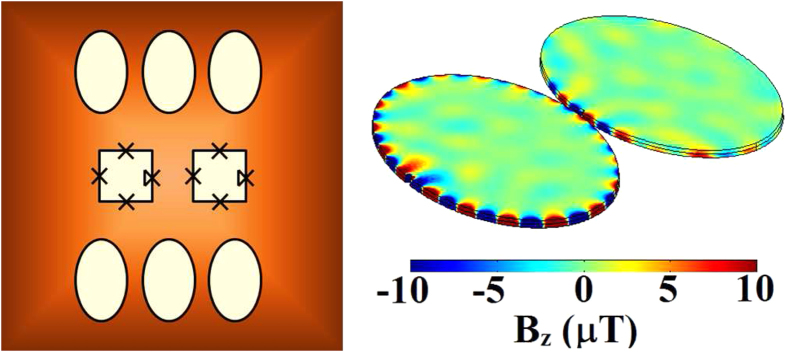
Whispering galleries and quantum devices. Left:the concept of transferral of whispering modes along linear arrays of elliptical disks, that are patterned in close proximity, relies on a layout such as that shown schematically here. The plasmonic devices are the outer ellipses. Between the two lines of ellipses are two *π*-rings, each consisting of three normal Josephson junctions and one *π*-junction. Right: Two microdisks each have an elliptical cone structure (major and minor lengths of 40 and 27 *μm*, respectively) with 10° semi-angles from the base. They consist of an underlayer of silver and a top layer of titanium dioxide (each 0.5 *μm* thick). The disks are separated by 0.8 *μm*. Each notch runs the full height of a disk and is 0.1 *μm* deep and wide.

**Figure 2:**
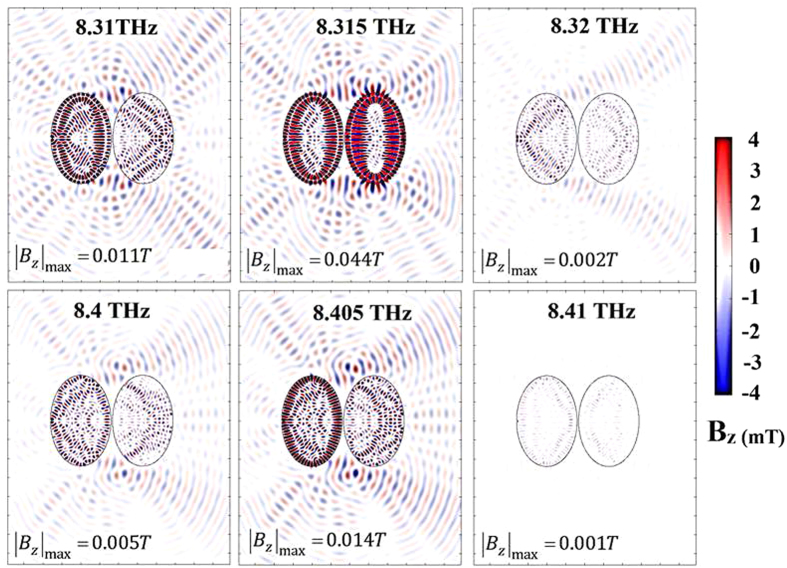
The electromagnetic field is focused at the centre of the left hand edge of the left elliptical disk, which contains a subwavelength sized notch. The propagation of the electric field can create a frequency dependent pumping of the system between whispering galleries. The maximum magnetic flux density in the *z*-direction is shown below the disks in each plot for the corresponding frequencies (shown above).

**Figure 3:**
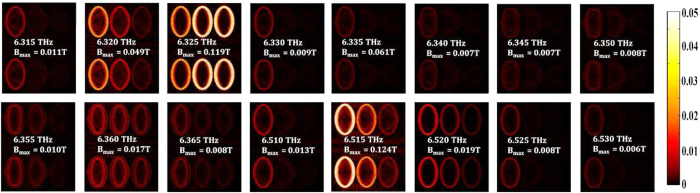
Switching over a range of 6.315 to 6.530 *THz* in a transverse electric field. The colour bar indicates the strength of the resulting magnetic flux density (limited to 0.05 T for greater visual clarity), which is much higher around the edges of the disks than at the locations of the π-rings. Whispering gallery modes of varying intensities appear around the elliptical disks as a function of frequency. The maximum field, *B*_*max*_ is indicated for each frequency.

**Figure 4:**
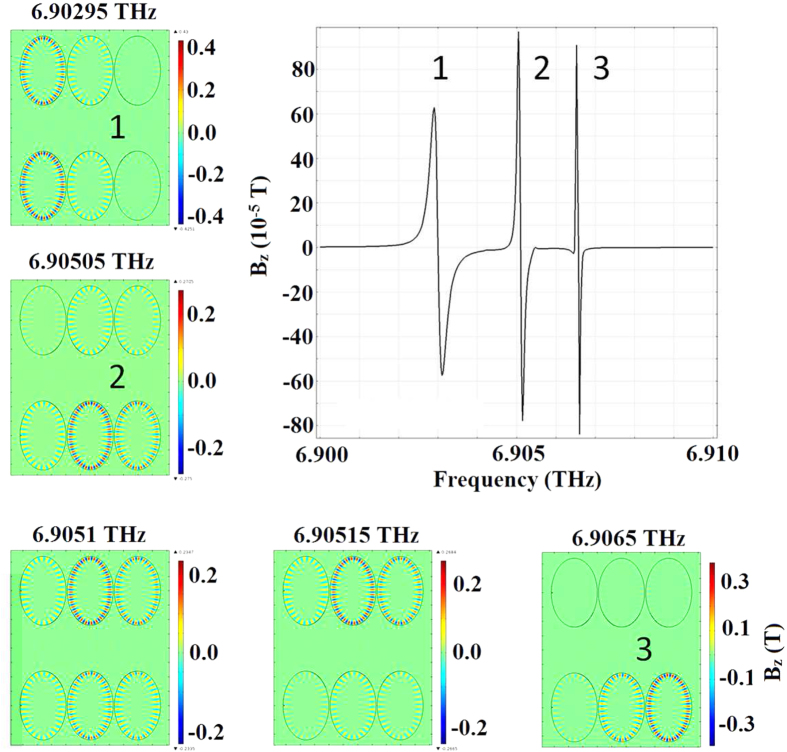
Microlaser switching over a range of frequencies close to 6.9 *THz* in a transverse electric field. The colour bar indicates the strength of the resulting magnetic flux density. The magnetic field *B*_*z*_ is a function of frequency and is measured at positions 1–3. The complex refractive indices of the disks are taken to be *N*_*TiO*2_ ≈ 2.025 + 0.500*i* and *N*_*Ag*_ = 6.168 + 44.787*i* at this frequency.

**Figure 5:**
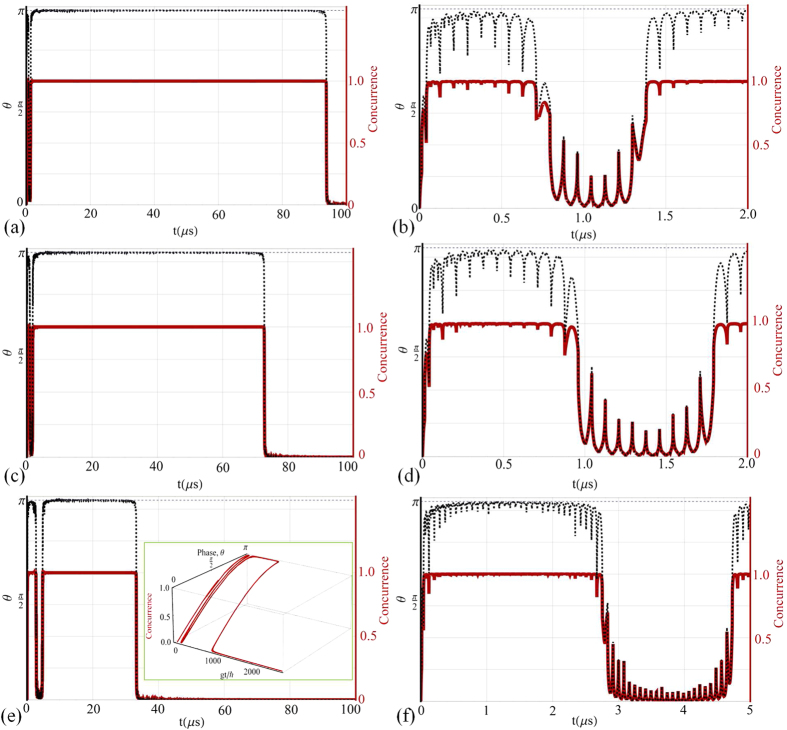
The concurrence for the system controlled by the elliptical cone array. Two 0.886 *μm* diameter circular *π*-rings are positioned as in Fig. 1. When the frequency is about 6.9 *THz*, and avoiding the frequencies that generate sharp resonance peaks in the controlling array (see Fig. 4), the local magnetic fields of the array are taken to be *B*_*p*1_ = 20 *μT* and *B*_*p*2_ = 70 *μT* in the *z*-direction. The qubit coupling in (**a,b**) is *g* = 2.80 × 10^−27^ *J*; (**c,d**) is *g* = 2.81 × 10^−27^ *J*; (**e,f**) is *g* = 2.85 × 10^−27^ *J*. The magnetic flux densities associated with the *π*-rings are *B*_*z*1,2_ ≈ 420 *μT* when there is half a flux quantum threading them. The inset of (**e**) shows the concurrence as both a function of phase *θ* and *gt*/*ħ*.

In the left-hand plasmonic disks in the systems of Figs 2, 3, and 4 there are small defects at each leading edge. These defects act to confine the field and then propagate all the modes. In this section we show the results of an entanglement analysis for this system when the applied frequency is ≈6.9 *THz* (as in Fig. 4). The Hamiltonian of the system is quite general^35^ and can be written as,




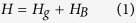







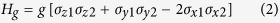







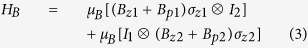




where *g* is the qubit coupling parameter and *μ*_*B*_ is the Bohr magneton. The spontaneous fields generated in the *π*-rings are *B*_*z*1,2_ (≈0.25 *μT* for 40 *μm* diameter rings, for example) and the fields extended by the plasmon waveguides to the vicinity of the qubits are *B*_*p*1,2_. The eigenvalues of this Hamiltonian are found to be, 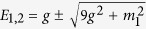 and 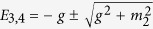 where, *m*_1,2_ = −*μ*_*B*_(*B*_*z*1_ + *B*_*p*1_ ± *B*_*z*2_ ± *B*_*p*2_). We consider the case where in addition to the plasmon wavequide fields *B*_*p*1,2_ there is a field in the *z*-direction that results from the spontaneous orbital moments of the *π*-rings. The two qubits evolve in time with a wavefunction of the form 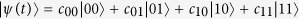. We now introduce a quantum phase gate that has the following action on the two qubit system: 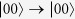; 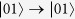; 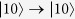; and 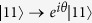. We are interested in creating a *π*-gate^35^ where the important phase, *θ* = ±*π*. To check the time evolution of the phase *θ* we need to solve the Schrödinger equation, in which the Hamiltonian is given by Eq. (1), and the time evolved wave function of the system leads us to the following form,




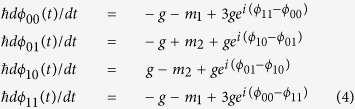




under the definition that 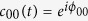, 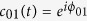, 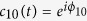, and 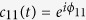. For this system *θ* = *ϕ*_11_ − *ϕ*_01_ − *ϕ*_10_ + *ϕ*_00_. We now show the evolution of the concurrence^36^,^37^ for the coupled *π*-ring qubits (see the Supplementary Information for a discussion of concurrence and its forms),




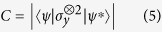




where normalisation is preserved throughout that maintains the monotonicity of the measure. The result is not dependent upon the initial conditions and here we chose *c*_00_(0) = *c*_11_(0) = 1/4 and 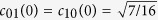. The plasmon waveguide has a series of whispering modes that propagate between the elliptical disks, with some disks switched “on” and others “off” at resonance. This is shown in Fig. 4 for the resonance peaks found at frequencies around ≈6.9 *THz*. Investigating the result of Fig. 4 one can see that the magnetic flux density between the two lines of the plasmon waveguide is around *B*_*p*_ ≈ 0.5 → 1.0 *mT* at resonance. The concurrence of two qubits located as in Fig. 1 for specific conditions is now found. One can see in the example of Fig. 5 that the concurrence can experience a series of entanglement sudden deaths and revivals. The phase gate can be made quite stable but as usual it is a case of finding the right energy balances in the system. For example, in Fig. 5 the system has a tendency to completely lose entanglement once before regaining *C* ≈ 1 for a duration until complete entanglement loss. By increasing the coupling strength between the *π*-rings the life-time of the phase gate actually becomes diminished. At *g* = 2.88 × 10^−27^ *J* no entanglement can be found at all. To find these regimes of *ESD* the energy balances of the system have to be suitably tuned and the fields from the controlling array can also strongly participate in the emergence of the phenomenon. For example, fixing the parameters of Fig. 5(e), except for *B*_*p*2_, to remain constant destroys entanglement when *B*_*p*2_ becomes less than 47 *μT*. The coupling between *π*-ring qubits, and indeed all genres of superconductive thin film rings^38^, is controllable through kinetic inductance and temperature at *THz* frequencies. Here we have taken a simple model based upon a parametric approach that allows one to circumvent the complicated issues of system-bath quantum dynamics to investigate a regime based upon the evolution of the Schrödinger equation where a phase gate can emerge. The adjustment of the magnetic parameters by small deviations reveals that the system can become highly unstable and *ESD* can even occur. Furthermore, for maximal entanglement and the creation of the phase gate, the qubits do not have to have the same dimensions when they are subjugated to the electromagnetic influence of the elliptical disks - which could be of high importance for designing qubits where dimensional tolerances are generally thought to have to be incredibly precise for them to work. The two qubit system can be extended to larger arrays and chains of elliptical disks used to control their entanglement.

## Discussion and Conclusions

Terahertz frequency plasmonic metamaterial devices (2–18 *THz*) have recently been made on flexible polypropylene substrates^39^. Plasmonic square apertures in gold films have also been fabricated and devices based on this arrangement have been shown to work effectively at *THz* frequencies^40^. Here, the terahertz fields are required in low temperature conditions so as to not destroy the superconductivity of the *π*-rings. The *π*-rings are a direct analogue to the split-ring resonators^41^ frequently found in metamaterials devices, with each ring having its own self-inductance and coupling through mutual inductance. The split gap is analogous to the Josephson junctions of the *π*-rings that provide a capacitance. The *π*-rings are exceptional in that they are low loss devices, that they generate their own orbital moments and have controllable magnetic permeability in a small external field^25^. This removes the requirement for additional circuitry to inject a current. The strength of the magnetic fields produced by the elliptical waveguide resonators is large enough to be able to switch the sign of the effective permeability of the rings from positive to negative (see^25^ for more details about the permeability of *π*-ring devices) when whispering galleries emerge as in Figs 3 and 4. It is with groups of naturally non-magnetic structures, metamaterials, that *THz* frequency waveguides can operate best, as the magnetic susceptibility drops off as one moves above microwave frequencies with conventional magnetic devices^42^. Indeed, incorporating non-magnetic materials as the controlling elements of plasmonic arrays leads to much larger magnetic field effects than could be achieved with natural magnetic materials^43^. Around the resonance frequencies of the plasmonic arrays the concurrence of the two qubits can fluctuate in a series of sudden deaths interspersed by rebirths. The phase gate can be switched on and off as a function of time and entanglement also exhibits a form of resonance. The results show that much like a sharp resonance peak, which occurs at a very precise frequency, rare coupled qubit energy balances can occasionally be found whereby the entanglement oscillates and for stable quantum computation these situations should be avoided or exploited with novel quantum algorithms. Capacitive coupling between two geometrically sensitive qubits has recently been discussed^44^ where one qubit is driven at its level-splitting with the other used to register the systems response. Likewise, the inductively or capacitively coupled *π*-ring qubits readout can be made by driving the left qubit with the plasmonic array, whilst the second qubit is used for detecting the coupling between them. In our case the use of the elliptical cone array can also modulate the coupling strength to produce a frequency dependent irregular dynamics in the system from which stability can be lost or regained.

In conclusion, we found a new method to control silent phase^45^ or flux qubits. The method is based on a formation of “whispering gallery” modes, created by a configuration of elliptic metallic disks that are subjected to electromagnetic radiation. For a long period of time, producing error-free entangling and controlling operations with superconducting qubits was a bottleneck for scalable quantum computation. Our results offer a scalable solution for these problems and demonstrate the advantages of the use of “whispering gallery” modes and the associated configurations of the metallic disks. The system is simple enough for immediate experimental realisation and designs can be adapted for specific purposes (such as quantum computation or communication); which is one of the advantages of using artificial atoms rather than natural ones^46^. We believe that our results will lead to rapid prototyping of these hybrid systems, leading to practical applications. The work constitutes an important step toward scalable superconductor quantum technologies based on coupled silent phase or flux qubits.

## Additional Information

**How to cite this article**: Forrester, D. M. and Kusmartsev, F. V. Whispering galleries and the control of artificial atoms. *Sci. Rep.* **6**, 25084; doi: 10.1038/srep25084 (2016).

## Supplementary Material

Supplementary Information
