# Supplementary material for: Whispering galleries and the control of artificial atoms
Source: Sci Rep. 2016 Apr 28;6:25084. doi: 10.1038/srep25084 (PMC4848508; doi:10.1038/srep25084)
Supplement: Supplementary Information [file srep25084-s1.pdf]

Whispering galleries and the control of artificial atoms: Supplementary Information

D. M. Forrester and F. V. Kusmartsev

Two artificial atoms

In order to allow efficient single-qubit and two-qubit operations (logic gates) control over the qubits Hamiltonian must be exhibited. This means that the magnetic fields at the locations of each qubit need to be controlled individually. The system has to be able to couple qubits together without detrimental consequences. The ideal set-up involves the ability to switch the coupling on and off. Thus the formation of superposition states could occur along with entanglement. The use of whispering galleries in microlasers provides a means with which to achieve these objectives. In Fig. S1 some possible schematics of control element/qubit architectures is shown.

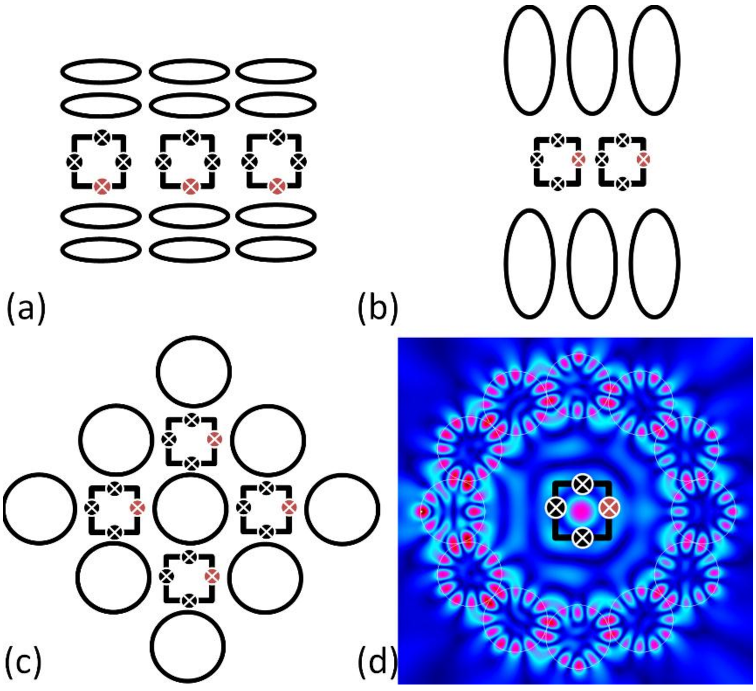

Figure S1: Different whispering gallery control element/qubit arrangements. The ellipses (open rings) propagate the whispering modes and manipulate the magnetic fields of the π-ring qubits (squares: each shown with three normal junctions (black) and one π-junction (red)).

The degree of entanglement occurs as a function of the interaction energies and temperature of the system. For example, if we write the system Hamiltonian to include dipole-dipole interaction and exchange interaction, it can take the form

$$\mathrm{H} = \varepsilon_A \sigma_{zA} + \varepsilon_B \sigma_{zB} + J \sigma_{zB} \otimes \sigma_{zB} + g\left(\sigma_{zA} \otimes \sigma_{zB} + \sigma_{yA} \otimes \sigma_{yB} - 2\sigma_{xA} \otimes \sigma_{xB}\right) \quad \text{(S1)}$$

from which a simple density matrix analysis can be formed . In equation S1 the energies of qubit A and B are contained in $\varepsilon_{A/B}$ inclusive of the energies of each π -ring and the control elements. For simplicity we will set $g = 0$ and demonstrate the sensitivity of concurrence even in a reduced Hamiltonian model. When the qubits begin to interact through the coupling term, the following table demonstrates the kind of eigenvalues and vectors that emerge.

Eigenvalue	Eigenvector
$-\sqrt{J^2 + \varepsilon_B{}^2 - 2\varepsilon_A\varepsilon_B + \varepsilon_A{}^2}$	$\begin{bmatrix}0 & \Lambda_1 & 1 & 0\end{bmatrix}^T$
$+\sqrt{J^2 + \varepsilon_B{}^2 - 2\varepsilon_A\varepsilon_B + \varepsilon_A{}^2}$	$\begin{bmatrix}0 & \Lambda_2 & 1 & 0\end{bmatrix}^T$
$-\sqrt{J^2 + \varepsilon_B{}^2 + 2\varepsilon_A\varepsilon_B + \varepsilon_A{}^2}$	$\begin{bmatrix}\Lambda_3 & 0 & 0 & 1\end{bmatrix}^T$
$+\sqrt{J^2 + \varepsilon_B{}^2 - 2\varepsilon_A\varepsilon_B + \varepsilon_B{}^2}$	$\begin{bmatrix}\Lambda_4 & 0 & 0 & 1\end{bmatrix}^T$

Table S1: Eigenvalues and eigenvectors of Hamiltonian (S1) with $g = 0$

In Table S1,

$$\begin{aligned}
\Lambda_1 &= \left(-\varepsilon_B + \varepsilon_A - \sqrt{J^2 + \varepsilon_B{}^2 - 2\varepsilon_A\varepsilon_B + \varepsilon_A{}^2}\right)\Big/ J \\
\Lambda_2 &= \left(-\varepsilon_B + \varepsilon_A + \sqrt{J^2 + \varepsilon_B{}^2 - 2\varepsilon_A\varepsilon_B + \varepsilon_A{}^2}\right)\Big/ J \\
\Lambda_3 &= \left(\varepsilon_B + \varepsilon_A - \sqrt{J^2 + \varepsilon_B{}^2 + 2\varepsilon_A\varepsilon_B + \varepsilon_A{}^2}\right)\Big/ J \\
\Lambda_4 &= \left(\varepsilon_B + \varepsilon_A + \sqrt{J^2 + \varepsilon_B{}^2 - 2\varepsilon_A\varepsilon_B + \varepsilon_B{}^2}\right)\Big/ J
\end{aligned} \quad \text{(S2)}$$

This leads to the following normalised equations through the fact that $\langle\psi|\psi\rangle = |a|^2 + |b|^2 = 1$ when $|\psi\rangle = a|\alpha\rangle + b|\beta\rangle$:

$$\begin{aligned}
|\psi_1\rangle &= \varsigma_1|10\rangle + \eta_1|01\rangle \\
|\psi_2\rangle &= \varsigma_2|10\rangle + \eta_2|01\rangle \\
|\psi_3\rangle &= \varsigma_3|00\rangle + \eta_3|11\rangle \\
|\psi_4\rangle &= \varsigma_4|00\rangle + \eta_4|11\rangle
\end{aligned} \quad \text{(S3)}$$

In equations (S3) the normalised quantities ς and η are representations equivalent to,

$$\varsigma = \frac{\Lambda}{\sqrt{\Lambda^2 + 1}} \quad \& \quad \eta = \frac{1}{\sqrt{\Lambda^2 + 1}}. \quad \text{(S4)}$$

To simplify the equations another transformation is made:

$$M=\sqrt{J^2+\varepsilon_B{}^2+2\varepsilon_A\varepsilon_B+\varepsilon_A{}^2}$$
$$N=\sqrt{J^2+\varepsilon_B{}^2-2\varepsilon_A\varepsilon_B+\varepsilon_B{}^2} \qquad \text{(S5)}$$

Eigenvalue	Distribution
-N	$P_1=\left(1+e^{-2\beta N}+e^{\beta(M-N)}+e^{-\beta(M+N)}\right)^{-1}$
N	$P_2=\left(1+e^{2\beta N}+e^{\beta(M+N)}+e^{\beta(N-M)}\right)^{-1}$
-M	$P_3=\left(1+e^{-2\beta M}+e^{\beta(N-M)}+e^{-\beta(M+N)}\right)^{-1}$
M	$P_4=\left(1+e^{2\beta M}+e^{\beta(N+M)}+e^{\beta(M-N)}\right)^{-1}$

Table S2: Probability distributions where $\beta=1/K_BT$

Now the values from the above table are to be introduced into the following form for the density matrix, $\rho_{AB}(T)=P_1|\psi_1\rangle\langle\psi_1|+P_2|\psi_2\rangle\langle\psi_2|+P_3|\psi_3\rangle\langle\psi_3|+P_4|\psi_4\rangle\langle\psi_4|$ to give

$$\rho_{AB}(T)=\begin{bmatrix} P_3\varsigma_3^2+P_4\varsigma_4^2 & 0 & 0 & P_3\varsigma_3\eta_3+P_4\varsigma_4\eta_4 \\ 0 & P_1\eta_1^2+P_2\eta_2^2 & P_1\varsigma_1\eta_1+P_2\varsigma_2\eta_2 & 0 \\ 0 & P_1\varsigma_1\eta_1+P_2\varsigma_2\eta_2 & P_1\varsigma_1^2+P_2\varsigma_2^2 & 0 \\ P_3\varsigma_3\eta_3+P_4\varsigma_4\eta_4 & 0 & 0 & P_3\eta_3^2+P_4\eta_4^2 \end{bmatrix} \qquad \text{(S6)}$$

and $\bar{\rho}_{AB}=\left(\sigma_{yA}\otimes\sigma_{yB}\right)\rho_{AB}^{*}\left(\sigma_{yA}\otimes\sigma_{yB}\right)$; with the concurrence found from the set of eigenvalues of $\rho_{AB}\,\bar{\rho}_{AB}$:

$$\{r\}=\left\{\begin{array}{l} 2\left[P_1^2\varsigma_1^2\eta_1^2+P_2\varsigma_2^2\eta_2^2\pm\sqrt{\left(P_1\varsigma_1^2+P_2\varsigma_2^2\right)\left(P_1\varsigma_1\eta_1+P_2\varsigma_2\eta_2\right)^2\left(P_1^2\eta_1^2+P_2\eta_2^2\right)}\right] \\ +P_1P_2\left(\varsigma_2\eta_1+\varsigma_1\eta_2\right)^2, \\ \\ 2\left[P_3^2\varsigma_3^2\eta_3^2+P_4\varsigma_4^2\eta_4^2\pm\sqrt{\left(P_3\varsigma_3^2+P_4\varsigma_4^2\right)\left(P_3\varsigma_3\eta_3+P_4\varsigma_4\eta_4\right)^2\left(P_3^2\eta_3^2+P_4\eta_4^2\right)}\right] \\ +P_3P_4\left(\varsigma_4\eta_3+\varsigma_3\eta_4\right)^2 \end{array}\right\} \qquad \text{(S7)}$$

For the Concurrence equation we require the square roots of the above eigenvalues ($\lambda_i=\sqrt{r_i}$). The concurrence[1] is found as the maximum of

$$C=\max\left(0;\lambda_1-\lambda_2-\lambda_3-\lambda_4\right) \qquad \text{(S8)}$$

with λ_i in decreasing order[1].

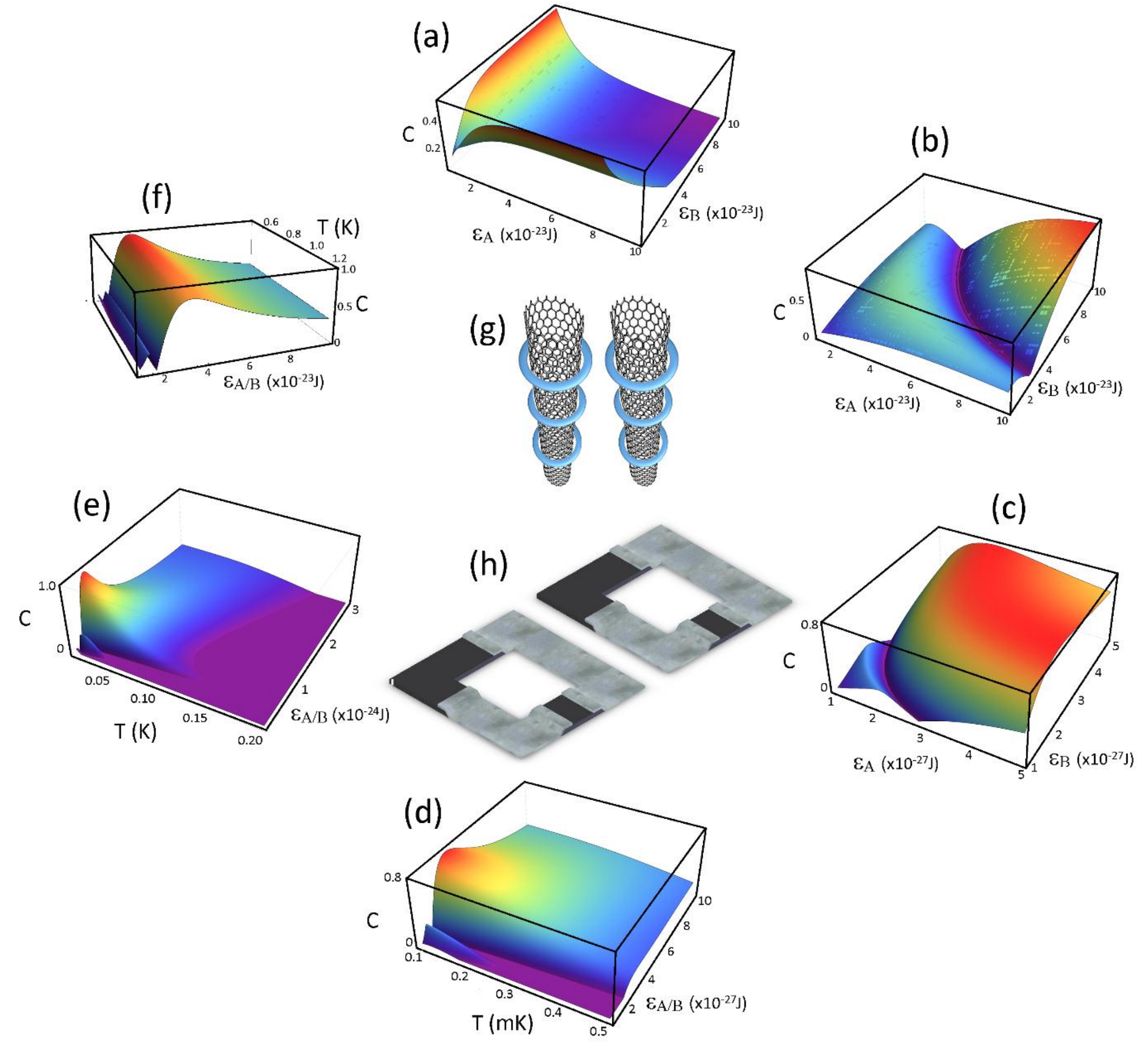

Figure S2: Concurrence as a function of temperature and qubit energy. (a) Nano-SQUID rings of $30\mathrm{nm}$ *diameter, at temperature* $\mathrm{T}=0.8\mathrm{K}$*, coupling energy* $\mathrm{J}=0.52\times10^{-23}\mathrm{J}$ *, and* $\varepsilon=1\rightarrow10\times10^{-23}\mathrm{J}$ *in each qubit. (b) Strongly coupled qubits with* $\mathrm{J}=11.24\times10^{-23}\mathrm{J}$ *but otherwise the same parameters as in (a). (c) Micro-*$\pi-\mathrm{rings}$ *of width* $3\mu\mathrm{m}$ *at* $\mathrm{T}=0.1\mathrm{mK}$ *,* $\varepsilon=1\rightarrow5\times10^{-27}\mathrm{J}$*, and* $\mathrm{J}=2.86\times10^{-27}\mathrm{J}$ *. (d) Same coupling energy as in (c) with* $\varepsilon_{\mathrm{A/B}}=1\rightarrow10\times10^{-27}\mathrm{J}$ *and* $\mathrm{T}=0.1\rightarrow0.5\mathrm{mK}$ *. (e)* $\varepsilon_{\mathrm{A/B}}=0.01\rightarrow3\times10^{-24}\mathrm{J}$*,* $\mathrm{J}=4.25\times10^{-25}\mathrm{J}$*, and* $\mathrm{T}=0.01\rightarrow0.2\mathrm{K}$*. (f)* $\varepsilon_{\mathrm{A/B}}=1\rightarrow10\times10^{-23}\mathrm{J}$*,* $\mathrm{J}=2.86\times10^{-23}\mathrm{J}$*, and* $\mathrm{T}=0.6\rightarrow1.2\mathrm{K}$*. (g) Superconducting* $\pi-\mathrm{rings}$ *around nanotubes. (h)* $\pi-\mathrm{ring}$ *qubits with ramp-Josephson junctions made from niobium and YBCO*[2]*.*

We will now show two scenarios for the concurrence: one with π-rings of micron-sized lengths, operating close to absolute zero, and another with nano-rings supported on carbon nanotube structures[3] or drawn quartz rods[4].

In Fig. S2 one can see that the maximum concurrence is highly dependent upon system temperature, the energy balances of the qubits, and their interactions. Indeed, in some regimes there is a very subtle energy balance been no entanglement and the occurrence of rapidly increasing concurrence gradients (as a function of temperature and $\varepsilon_{\mathrm{A/B}}$). Thus, a density matrix analysis supports the possibility of

finding entanglement sudden death near critical energy levels in the system. Indeed, fluctuations in temperature could even push the system to obtain rapid fluctuations in concurrence.

Entanglement of Formation

The entanglement of formation is an entanglement monotone given by

$$E_F(\rho) = \min \sum_i p_i E(\psi_i), \quad \text{(S9)}$$

where $E(\psi_i) = S(\rho(A)_i)$ and

$S(\rho(A)) = -\mathrm{Tr}(\rho(A)\log_2 \rho(A)) = -\mathrm{Tr}(\rho(B)\log_2 \rho(B))$ is the von-Neumann entropy (and A and B represent qubit 1 and qubit 2, respectively). The minimum is taken over all the possible realisations of the state

$$\rho(AB) = \sum_j p_j |\psi_j\rangle\langle\psi_j| \text{ with } \rho(A)_i = \mathrm{Tr}_B(|\psi_i\rangle\langle\psi_i|) \quad \text{(S10)}$$

A measure of entanglement should be zero for any separable state, E = 0. I.e. in a pure state the entropy is zero. The von-Neumann entropy is used to look at classical and quantum correlations (the classical Shannon entropy can only be used for the macroscopic world). It is invariant under unitary transformations and as such can be used to give a total picture of the entropy of a system.

$$S(U\rho U^{\dagger}) = S(\rho) \quad \text{(S11)}$$

Example: if U is the Pauli-x matrix (which is unitary, i.e. $U^{\dagger}U = I$) and the pure state is $|\psi\rangle = |00\rangle$

$$\rho = |\psi\rangle\langle\psi| = \begin{bmatrix} 1 & 0 & 0 & 0 \\ 0 & 0 & 0 & 0 \\ 0 & 0 & 0 & 0 \\ 0 & 0 & 0 & 0 \end{bmatrix} \quad \text{(S12)}$$

Now performing the transformation,

$$U\rho U^{\dagger} = \begin{bmatrix} 0 & 0 & 0 & 0 \\ 0 & 0 & 0 & 0 \\ 0 & 0 & 1 & 0 \\ 0 & 0 & 0 & 0 \end{bmatrix} \quad \text{(S13)}$$

It can easily be seen that the entropy has remained constant throughout the process:

$$S(U\rho U^{\dagger}) = S(\rho) = 1\log_2 1 = 0 \quad \text{(S14)}$$

The entanglement of a two qubit pure state can be interpreted by using the following in the computational basis

$$|\psi\rangle = a|00\rangle + b|10\rangle + c|01\rangle + d|11\rangle \quad \text{(S15)}$$

Here a, b, c, and d are complex numbers that will equate to $|a|^2 + |b|^2 + |c|^2 + |d|^2 = 1$. The density matrix of this two-qubit system is then,

$$\rho(AB) = |\psi\rangle\langle\psi| = (a|00\rangle + b|10\rangle + c|01\rangle + d|11\rangle)(a^*\langle 00| + b^*\langle 10| + c^*\langle 01| + d^*\langle 11|) =$$

$$aa^*|00\rangle\langle 00| + ab^*|00\rangle\langle 10| + ac^*|00\rangle\langle 01| + ad^*|00\rangle\langle 11|$$

$$+ba^*|10\rangle\langle 00| + bb^*|10\rangle\langle 10| + bc^*|10\rangle\langle 01| + bd^*|10\rangle\langle 11|$$

$$+ca^*|01\rangle\langle 00| + cb^*|01\rangle\langle 10| + cc^*|01\rangle\langle 01| + cd^*|01\rangle\langle 11|$$

$$+da^*|11\rangle\langle 00| + db^*|11\rangle\langle 10| + dc^*|11\rangle\langle 01| + dd^*|11\rangle\langle 11|$$

This written in matrix form is,

$$\rho(AB)=\begin{pmatrix} |a|^2 & ba^* & ca^* & da^* \\ ab^* & |b|^2 & cb^* & db^* \\ ac^* & bc^* & |c|^2 & dc^* \\ ad^* & bd^* & cd^* & |d|^2 \end{pmatrix} \tag{S16}$$

From which,

$$\rho(A)=\begin{pmatrix} \mathrm{Tr}\begin{pmatrix} |a|^2 & ba^* \\ ab^* & |b|^2 \end{pmatrix} & \mathrm{Tr}\begin{pmatrix} ca^* & da^* \\ cb^* & db^* \end{pmatrix} \\ \mathrm{Tr}\begin{pmatrix} ac^* & bc^* \\ ad^* & bd^* \end{pmatrix} & \mathrm{Tr}\begin{pmatrix} |c|^2 & dc^* \\ cd^* & |d|^2 \end{pmatrix} \end{pmatrix}=\begin{pmatrix} |a|^2+|b|^2 & ca^*+db^* \\ ac^*+bd^* & |c|^2+|d|^2 \end{pmatrix} \tag{S17}$$

The determinant, D, of the above is

$$\begin{aligned} D &\equiv \det(\rho(A))=\left(|a|^2+|b|^2\right)\left(|c|^2+|d|^2\right)-\left(ca^*+db^*\right)\left(ac^*+bd^*\right) \\ &=|ac|^2+|ad|^2+|bc|^2+|bd|^2-|ac|^2-a^*bcd^*-ab^*c^*d-|bd|^2 \\ &=(ad-bc)(ad-bc)^* \\ &=|ad-bc|^2 \end{aligned} \tag{S18}$$

The eigenvalues are found below,

$$\begin{aligned} &\det(\rho(A)-\lambda I)=0 \\ &=\left(|a|^2+|b|^2-\lambda\right)\left(|c|^2+|d|^2-\lambda\right)-\left(ca^*+db^*\right)\left(ac^*+bd^*\right)=0 \\ &=|ac|^2+|ad|^2-\lambda|a|^2+|bc|^2+|bd|^2-\lambda|b|^2-\lambda|c|^2+\lambda^2-\lambda|d|^2-|ac|^2-a^*bcd^*-ab^*c^*d-|bd|^2=0 \\ &=\lambda^2-\left(|a|^2+|b|^2+|c|^2+|d|^2\right)\lambda+\left(|ad|^2+|bc|^2-a^*bcd^*-ab^*c^*d\right)=0 \\ &=\lambda^2-\lambda+|ad-bc|^2 \end{aligned}$$

Therefore the eigenvalues are,

$$\lambda=\left(1\pm\sqrt{1-4|ad-bc|^2}\right)/2 \tag{S19}$$

From this the entropy is

$$\begin{aligned} S(\rho(A))=-\sum\lambda\log_2\lambda=&-\left(\left(1+\sqrt{1-4D}\right)/2\right)\log_2\left(\left(1+\sqrt{1-4D}\right)/2\right) \\ &-\left(\left(1-\sqrt{1-4D}\right)/2\right)\log_2\left(\left(1-\sqrt{1-4D}\right)/2\right) \end{aligned} \tag{S20}$$

Saying that $p=\left(1-\sqrt{1-4D}\right)/2$ allows this to be written as the binary entropy function as,

$$(1-p)=1-\left(1-\sqrt{1-4D}\right)/2=\left(1+\sqrt{1-4D}\right)/2 \tag{S21}$$

This gives,

$$H(p,1-p)=-p\log_2 p-(1-p)\log_2(1-p) \tag{S22}$$

The entanglement for a pure state is

$$E(\psi)=S(\rho(A))=-p\log_2 p-(1-p)\log_2(1-p) \tag{S23}$$

A new basis called the magic basis is now stated[1,5]. This is basically the Bell states subjected to some phase shifting.

$$
\begin{aligned}
|e_1\rangle &= \frac{1}{\sqrt{2}}(|00\rangle + |11\rangle) \\
|e_2\rangle &= \frac{i}{\sqrt{2}}(|00\rangle - |11\rangle) \\
|e_3\rangle &= \frac{i}{\sqrt{2}}(|01\rangle + |10\rangle) \\
|e_4\rangle &= \frac{1}{\sqrt{2}}(|01\rangle - |10\rangle)
\end{aligned} \tag{S24}
$$

(E.g. for each of the states

$|e_2\rangle = i(\sigma_z \otimes I)|e_1\rangle, |e_3\rangle = i(\sigma_x \otimes I)|e_1\rangle$ and $|e_4\rangle = i(\sigma_y \otimes I)|e_1\rangle$) (S25)

Expanding the two qubit pure state $|\psi\rangle$ in the magic basis[1,5]:

$$
|\psi\rangle = \sum_i^4 \alpha_i |e_i\rangle = \alpha_1|e_1\rangle + \alpha_2|e_2\rangle + \alpha_3|e_3\rangle + \alpha_4|e_4\rangle \tag{S26}
$$

In this α_i are complex numbers and $\sum_i |\alpha_i|^2 = 1$. The values of a, b, c, and d, in terms of alpha, can at this instant be found:

$$
\begin{aligned}
&\alpha_1|e_1\rangle + \alpha_2|e_2\rangle + \alpha_3|e_3\rangle + \alpha_4|e_4\rangle = a|00\rangle + b|10\rangle + c|01\rangle + d|11\rangle \\
&\Rightarrow 1/\sqrt{2}\left((\alpha_1 + \alpha_2 i)|00\rangle + (-\alpha_4 + \alpha_3 i)|10\rangle + (\alpha_3 i + \alpha_4)|01\rangle + (\alpha_1 - \alpha_2 i)|11\rangle\right) \\
&= a|00\rangle + b|10\rangle + c|01\rangle + d|11\rangle
\end{aligned} \tag{S27}
$$

Consequently,

$$
\begin{aligned}
a &= (\alpha_1 + \alpha_2 i)/\sqrt{2} \\
b &= (-\alpha_4 + \alpha_3 i)/\sqrt{2} \\
c &= (\alpha_3 i + \alpha_4)/\sqrt{2} \\
d &= (\alpha_1 - \alpha_2 i)/\sqrt{2}
\end{aligned} \tag{S28}
$$

Thus giving,

$$
ad - bc = \frac{(\alpha_1{}^2 + \alpha_2{}^2 + \alpha_3{}^2 + \alpha_4{}^2)}{2} \tag{S29}
$$

The concurrence[5] is defined by $C = \left|\sum_i \alpha_i{}^2\right|$ and so $C = 2|ad - bc|$. This means that $D = C^2/4$ and the entanglement of formation can be rewritten for concurrence:

$$
\begin{aligned}
E(\psi) = &-\left(\left(1+\sqrt{1-C^2}\right)/2\right)\log_2\left(\left(1+\sqrt{1-C^2}\right)/2\right) \\
&-\left(\left(1-\sqrt{1-C^2}\right)/2\right)\log_2\left(\left(1-\sqrt{1-C^2}\right)/2\right)
\end{aligned} \tag{S30}
$$

The Spin-Flip

The concurrence is defined in terms of the spin-flip operation. For a single qubit the spin-flipped density operator is given by

$$
\tilde{\rho} = \sigma_y \rho^* \sigma_y \tag{S31}
$$

Written as $\rho = \frac{1}{2}(\mathrm{I} + \mathbf{P}.\boldsymbol{\sigma})$ with $\mathbf{P} = (\mathrm{Cos}(\varphi)\mathrm{Sin}(\theta), \mathrm{Sin}(\varphi)\mathrm{Sin}(\theta), \mathrm{Cos}(\theta))$ and σ being the Pauli matrices the density matrix becomes,

$$\rho = \frac{1}{2}\begin{pmatrix} 1+\mathrm{Cos}(\theta) & \mathrm{Cos}(\varphi)\mathrm{Sin}(\theta) - \mathrm{iSin}(\varphi)\mathrm{Sin}(\theta) \\ \mathrm{Cos}(\varphi)\mathrm{Sin}(\theta) + \mathrm{iSin}(\varphi)\mathrm{Sin}(\theta) & 1-\mathrm{Cos}(\theta) \end{pmatrix} \quad \text{(S32)}$$

The Complex conjugate of this is

$$\rho = \frac{1}{2}\begin{pmatrix} 1+\mathrm{Cos}(\theta) & \mathrm{Cos}(\varphi)\mathrm{Sin}(\theta) + \mathrm{iSin}(\varphi)\mathrm{Sin}(\theta) \\ \mathrm{Cos}(\varphi)\mathrm{Sin}(\theta) - \mathrm{iSin}(\varphi)\mathrm{Sin}(\theta) & 1-\mathrm{Cos}(\theta) \end{pmatrix} \quad \text{(S33)}$$

The spin-flip for a single qubit – given the above definition – is

$$\tilde{\rho} = \frac{1}{2}\begin{pmatrix} 1-\mathrm{Cos}(\theta) & -\mathrm{Cos}(\varphi)\mathrm{Sin}(\theta) + \mathrm{iSin}(\varphi)\mathrm{Sin}(\theta) \\ -\mathrm{Cos}(\varphi)\mathrm{Sin}(\theta) - \mathrm{iSin}(\varphi)\mathrm{Sin}(\theta) & 1+\mathrm{Cos}(\theta) \end{pmatrix} \quad \text{(S34)}$$

This simply takes the form,

$$\tilde{\rho} = \frac{1}{2}(\mathrm{I} - \mathbf{P}.\boldsymbol{\sigma}) \quad \text{(S35)}$$

Example: If we put $\varphi = 0$ and $\theta = \pi/2$ then $\rho = \begin{pmatrix} 0.5 & 0.5 \\ 0.5 & 0.5 \end{pmatrix}$ and $\tilde{\rho} = \begin{pmatrix} 0.5 & -0.5 \\ -0.5 & 0.5 \end{pmatrix}$. With a little working it can be seen that

$|\psi\rangle = (|0\rangle + |1\rangle)/\sqrt{2}$ and $|\tilde{\psi}\rangle = (|0\rangle - |1\rangle)/\sqrt{2}$

This can be visualised in the Bloch picture as

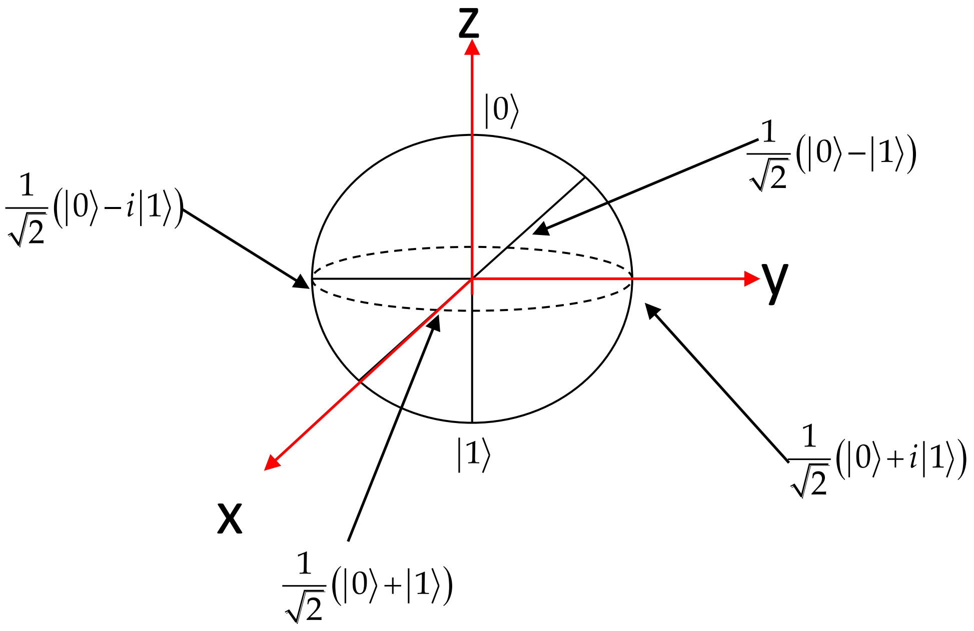

Figure S3: The Bloch sphere and the various representations for each axis.

For two qubits the spin-flip is the following operation,

$$|\tilde{\psi}_{AB}\rangle = (\sigma_y{}^A \otimes \sigma_y{}^B)|\psi^*{}_{AB}\rangle$$
$$\langle\tilde{\psi}_{AB}| = \langle\psi^*{}_{AB}|(\sigma_y{}^A \otimes \sigma_y{}^B) \quad \text{(S36)}$$

Hence, the density matrix is

$$\tilde{\rho}=|\tilde{\psi}_{AB}\rangle\langle\tilde{\psi}_{AB}|=\left(\sigma_y{}^A\otimes\sigma_y{}^B\right)|\psi^*{}_{AB}\rangle\langle\psi^*{}_{AB}|\left(\sigma_y{}^A\otimes\sigma_y{}^B\right)$$
$$=\left(\sigma_y{}^A\otimes\sigma_y{}^B\right)\rho^*\left(\sigma_y{}^A\otimes\sigma_y{}^B\right) \tag{S37}$$

We have already stated that the density matrix in terms of a-d can be written as,

$$\rho(AB)=\begin{pmatrix} aa^* & ba^* & ca^* & da^* \\ ab^* & bb^* & cb^* & db^* \\ ac^* & bc^* & cc^* & dc^* \\ ad^* & bd^* & cd^* & dd^* \end{pmatrix} \tag{S38}$$

So,

$$\rho^*(AB)=\begin{pmatrix} a^*a & a^*b & a^*c & a^*d \\ b^*a & b^*b & b^*c & b^*d \\ c^*a & c^*b & c^*c & c^*d \\ d^*a & d^*b & d^*c & d^*d \end{pmatrix} \tag{S39}$$

and,

$$\tilde{\rho}=\left(\sigma_y{}^A\otimes\sigma_y{}^B\right)\rho^*\left(\sigma_y{}^A\otimes\sigma_y{}^B\right)=\begin{pmatrix} d^*d & -c^*d & -b^*d & a^*d \\ -d^*c & c^*c & b^*c & -a^*c \\ -d^*b & c^*b & b^*b & -a^*b \\ d^*a & -c^*a & -b^*a & a^*a \end{pmatrix}$$

(S40)

$$\rho\tilde{\rho}=\begin{pmatrix} aa^* & ba^* & ca^* & da^* \\ ab^* & bb^* & cb^* & db^* \\ ac^* & bc^* & cc^* & dc^* \\ ad^* & bd^* & cd^* & dd^* \end{pmatrix}\begin{pmatrix} d^*d & -c^*d & -b^*d & a^*d \\ -d^*c & c^*c & b^*c & -a^*c \\ -d^*b & c^*b & b^*b & -a^*b \\ d^*a & -c^*a & -b^*a & a^*a \end{pmatrix} \tag{S41}$$

$$Tr(\rho\tilde{\rho})=4\left(aa^*dd^*-a^*bcd^*-ab^*c^*d+bb^*cc^*\right)$$
$$=4(ad-bc)\left(a^*d^*-b^*c^*\right) \tag{S42}$$

$$\sqrt{Tr(\rho\tilde{\rho})}=\sqrt{4(ad-bc)\left(a^*d^*-b^*c^*\right)}=2\sqrt{(ad-bc)\left(a^*d^*-b^*c^*\right)}$$
$$=2|ad-bc| \tag{S43}$$

This is the same as the previous definition for the concurrence of a pure state. The concurrence can also be written as,

$$C(\psi)=\sqrt{Tr(\rho\tilde{\rho})}=\sqrt{\langle\psi|(\sigma_y\otimes\sigma_y)\rho^*(\sigma_y\otimes\sigma_y)|\psi\rangle}$$
$$=\sqrt{\langle\psi|(\sigma_y\otimes\sigma_y)|\psi^*\rangle\langle\psi^*|(\sigma_y\otimes\sigma_y)|\psi\rangle}=\sqrt{\left|\langle\psi|(\sigma_y\otimes\sigma_y)|\psi^*\rangle\right|^2} \quad \text{(S44)}$$
$$=\left|\langle\psi|(\sigma_y\otimes\sigma_y)|\psi^*\rangle\right|=|\langle\psi|\tilde{\psi}\rangle|$$

Mixed State Concurrence

An important notational device in the following derivation is that of what has been termed "subnormalisation". If we write the density matrix as

$$\rho=\sum_j^m p_j\left|w_j\right\rangle\left\langle w_j\right|=\sum_j^n\left|\bar{w}_j\right\rangle\left\langle\bar{w}_j\right| \quad \text{(S45)}$$

then $\left|\bar{w}_j\right\rangle=\sqrt{p_j}\left|w_j\right\rangle$ are the subnormalised eigenvectors in an m-dimensional Hilbert space. The mixed state concurrence is derived by Wootters in terms of his "pure state decomposition theorem"[1]. This theorem tells us that a set of subnormalised vectors gives a decomposition of the density matrix only if the vectors can be represented as

$$\left|\varpi_j\right\rangle=\sum_k^n M_{kj}\left|\bar{v}_k\right\rangle \qquad j=1,\ldots\ldots,m \quad \text{(S46)}$$

Here M_{kj} is any n x m matrix (can be made m x m by adding zero's in extra rows) where there exists n rows of orthonormal eigenvectors in m-dimensional space ($m\geq n$) and the set $\{|\bar{v}_1\rangle,\ldots\ldots,|\bar{v}_k\rangle\}$ is an orthonormal basis of eigenvectors that belong to eigenvalues $\lambda_1,\ldots\ldots,\lambda_k$. This set is called an eigen-ensemble of ρ where $\langle\bar{v}_k|\bar{v}_k\rangle=\lambda_k$. The density matrix of $\{|\bar{v}_1\rangle,\ldots\ldots,|\bar{v}_m\rangle\}$ is,

$$\sum_{j=1}^m\left|\bar{w}_j\right\rangle\left\langle\bar{w}_j\right|=\sum_{j=1}^m\sum_{i,k=1}^n\left(M_{ji}^*\right)M_{jk}|v_k\rangle\langle v_i| \quad \text{(S47)}$$

The sum over the M-matrices can be written as $\sum_{j=1}^m M_{ji}{}^*M_{jk}=\delta_{ik}$ so that,

$$\sum_{i,k=1}^n\delta_{ik}|\bar{v}_k\rangle\langle\bar{v}_i|\overset{\text{when}\, i=k}{=}\sum_k^n|\bar{v}_k\rangle\langle\bar{v}_k|=\rho \quad \text{(S48)}$$

In the above the orthonormality of the columns of k is used. From (S45) it is seen that

$$\left|\bar{w}_j\right\rangle=\sqrt{p_j}\left|w_j\right\rangle=\rho^{1/2}\left|w_j\right\rangle \quad \text{(S49)}$$

It follows that

$$\sum_{j=1}^m\rho^{-1/2}\left|\bar{w}_j\right\rangle\left\langle\bar{w}_j\right|\rho^{-1/2}=\sum_{j=1}^m\left|w_j\right\rangle\left\langle w_j\right| \quad \text{(S50)}$$

$$\rho^{-1/2}\left|\bar{w}_j\right\rangle=\sum_{k=1}^n U_{kj}|w_k\rangle \qquad j=1,\ldots\ldots,m \quad \text{(S51)}$$

I.e. the set of columns of M_{kj} can be extended to an orthonormal basis of $\mathbb{C}^m$ so that (S46) can be written as

$$\begin{pmatrix} |\bar{\omega}_1\rangle \\ \vdots \\ \vdots \\ \vdots \\ |\bar{\omega}_m\rangle \end{pmatrix} = U \begin{pmatrix} |\bar{v}_1\rangle \\ \vdots \\ |\bar{v}_n\rangle \\ |0\rangle \\ \vdots \\ |0\rangle \end{pmatrix} \quad \text{(S52)}$$

By adding additional $|0\rangle$'s for $k = n+1,......,m$, m orthonormal vectors in the extended Hilbert space are defined (U is an m x m unitary matrix),

$$|\hat{w}_j\rangle = \sum_{k=1}^{m} U_{kj} |w_k\rangle \qquad j = 1,......,m \quad \text{(S53)}$$

In this equation

$$|\bar{w}_j\rangle = \rho^{1/2} |\hat{w}_j\rangle \quad \text{(S54)}$$

For the two qubit state where $\rho = \sum_j^m p_j |w_j\rangle\langle w_j| = \sum_j^n |\bar{w}_j\rangle\langle \bar{w}_j|$ the average concurrence is

$$\langle C\rangle = \sum_j p_j C(w_j) = \sum_j p_j \left|\langle w_j|\sigma_y \otimes \sigma_y |w^*{}_j\rangle\right| \geq |\langle c\rangle| = \left|\sum_j p_j c(w_j)\right| \quad \text{(S55)}$$

Here $c(w_j)$ is the preconcurrence[1]. The preconcurrence is the concurrence without the absolute value signs that make the concurrence positive

$$c(w_j) = \langle w_j|\sigma_y \otimes \sigma_y |w^*{}_j\rangle \quad \text{(S56)}$$

The average preconcurrence is

$$\langle c\rangle = \sum_j \langle \bar{w}_j|\sigma_y \otimes \sigma_y |\bar{w}_j^*\rangle = \sum_j \langle \hat{w}_j|\rho^{1/2}(\sigma_y \otimes \sigma_y)(\rho^*)^{1/2}|\hat{w}_j^*\rangle = \mathrm{Tr}(\tau) \quad \text{(S57)}$$

$$\tau = \rho^{1/2}(\sigma_y \otimes \sigma_y)(\rho^*)^{1/2} \quad \text{(S58)}$$

It is of note that τ is a symmetric operator:

$$\begin{aligned} \tau_{ij} &= \langle \bar{w}_i|\sigma_y \otimes \sigma_y |\bar{w}_j^*\rangle = \left(\langle \bar{w}_j^*|\sigma_y \otimes \sigma_y |\bar{w}_i\rangle\right)^* \\ &= \langle \bar{w}_j|\sigma_y \otimes \sigma_y |\bar{w}_i^*\rangle = \tau_{ji} \end{aligned} \quad \text{(S59)}$$

Singular value decomposition and concurrence

From (S59) it can be seen that $\tau = \tau^T$ and the singular value decomposition can we expressed as

$$\tau = VSW^{*T} = \tau^T = W^* S V^T \quad \text{(S60)}$$

where $\tau = mxn$, $V = mxm$, $W = nxn$ and S is a diagonal matrix .

As τ is actually symmetric $m = n$ and the above shows that $W^{*T} = V^T$ so that

$$\tau = VSV^T \quad \text{(S61)}$$

So, V diagonalises τ and the elements of S are the singular values of τ, $S_{kl} = \lambda_k \delta_{kl}$.

The singular values of τ are the non-negative square roots of the eigenvalues of

$$\tau\tau^{*T} = \left(\rho^{1/2}\left(\sigma_y \otimes \sigma_y\right)\left(\rho^*\right)^{1/2}\right)\left(\rho^{1/2}\left(\sigma_y \otimes \sigma_y\right)\left(\rho^*\right)^{1/2}\right)^{*T} \quad \text{(S62)}$$

From $\tau = \tau^T$,

$$\begin{aligned}\tau\tau^{*T} &= \left(\rho^{1/2}\left(\sigma_y \otimes \sigma_y\right)\left(\rho^*\right)^{1/2}\right)\left(\rho^{1/2}\left(\sigma_y \otimes \sigma_y\right)\left(\rho^*\right)^{1/2}\right)^{*} \\ &= \left(\rho^{1/2}\left(\sigma_y \otimes \sigma_y\right)\left(\rho^*\right)^{1/2}\right)\left(\left(\rho^*\right)^{1/2}\left(\sigma_y \otimes \sigma_y\right)\rho^{1/2}\right) \\ &= \rho^{1/2}\left(\sigma_y \otimes \sigma_y\right)\rho^*\left(\sigma_y \otimes \sigma_y\right)\rho^{1/2}\end{aligned} \quad \text{(S63)}$$

The singular value decomposition can be employed as follows,

$$\begin{aligned}\langle C\rangle &= \sum_j \left|\tau_{jj}\right| = \sum_j \left|\sum_{kl} V_{jk} S_{kl} V_{jl}\right| = \sum_j \left|\sum_{kl} V_{jk}\lambda_k \delta_{kl} V_{jl}\right| \\ &= \sum_j \left|\sum_k V^2{}_{jk}\lambda_k\right| = \sum_j \left|V^2{}_{j1}\lambda_1 + \sum_{k\geq 1} V^2{}_{jk}\lambda_k\right|\end{aligned} \quad \text{(S64)}$$

Using the triangle inequality $|x|-|y| \leq |x+y| \leq |x|+|y|$,

$$\begin{aligned}\sum_j \left|V^2{}_{j1}\lambda_1 + \sum_{k\geq 1} V^2{}_{jk}\lambda_k\right| &\geq \sum_j \left|V^2{}_{j1}\lambda_1\right| - \left|\sum_{k\geq 1} V^2{}_{jk}\lambda_k\right| \\ &= \lambda_1 \sum_j \left|V^2{}_{j1}\right| - \sum_{k\geq 1}\lambda_k \left(\sum_j \left|V^2{}_{jk}\right|\right)\end{aligned} \quad \text{(S65)}$$

As $\sum_j \left|V^2{}_{jk}\right| = 1$ (S66)

$$\sum_j \left|V^2{}_{j1}\lambda_1 + \sum_{k\geq 1} V^2{}_{jk}\lambda_k\right| = \lambda_1 - \sum_{k\geq 1}\lambda_k = \lambda_1 - \lambda_2 - \lambda_3 - \lambda_4 = C(\rho) \quad \text{(S67)}$$

Wootters[1] has proved that using a series of decompositions that distribute the average preconcurrence that each member of the ensemble has equality with $\lambda_1 - \lambda_2 - \lambda_3 - \lambda_4$ or 0. Thus the equation is,

$$C(\rho) = \max\{0, \lambda_1 - \lambda_2 - \lambda_3 - \lambda_4\} \quad \text{(S68)}$$

Thus, we have shown the concurrence measure as defined by Wootters in a number of forms. In the main text the form of equation (5) is equivalent to that given in Ref. 1 (see derivation leading to equation S44), with normalisation included throughout in order to maintain monotonicity.

1. Wootters, W. K. Entanglement of formation of an arbitrary state of two qubits. Phys. Rev.Lett. 80 (10), 2245 (1998).
2. Kirtley, J. R. et al. Anti-ferromagnetic ordering in arrays of superconducting π-rings. Phys. Rev. B **72** (21), 214521 (2005).
3. Cleuziou, J.-P. et al. Carbon nanotube superconducting quantum interference device. *Nature Nanotechnology* **1**, 53 - 59 (2006).
4. Finkler, A. et al. Self-Aligned Nanoscale SQUID on a Tip. *Nano Lett*. **10** (3), 1046–1049 (2010).
5. Hill, S. & Wooters, W. K. Entanglement of a pair of quantum bits. Phys. Rev. Lett. 78(26), 5022 (1997).
